# mGem: Innate immune sensing of HIV-1 transcription in myeloid cells—a tale of two transcription start sites

**DOI:** 10.1128/mbio.03340-25

**Published:** 2026-10-20

**Authors:** Ivy K. Hughes, Suryaram Gummuluru

**Affiliations:** 1 Department of Virology, Immunology, & Microbiology, Boston University Chobanian & Avedisian School of Medicine 1846, Boston, Massachusetts, USA; Albert Einstein College of Medicine, Bronx, New York, USA

**Keywords:** human immunodeficiency virus, innate immunity, RNA sensing, chronic inflammation

## Abstract

Suppressive antiretroviral therapy (ART) in people with HIV (PWH) arrests progression to AIDS and onward viral transmission. However, viral transcription from integrated HIV-1 proviruses persists on treatment and is linked to markers of chronic immune activation, contributing to the accelerated development of comorbidities in PWH. Of particular interest are persistent responses in long-lived tissue-resident myeloid cells, which direct tissue-level inflammatory processes through cytokine secretion and interactions with other immune cell populations. Here, we review recent findings on viral nucleic acid sensing events in HIV-infected myeloid cells to shed light on possible mechanisms of persistent inflammation resulting from chronic HIV-1 transcription in myeloid cells in PWH.

## PERSPECTIVE

While antiretroviral therapy (ART) typically achieves durable suppression of HIV-1 replication and viremia in people with HIV (PWH), various negative health outcomes are still observed in PWH relative to their age-matched HIV-negative counterparts, which are likely to be at least partially due to persistent immune activation in PWH even under conditions of ART suppression (1). Importantly, immune activation synergizes with aging-related inflammation in many PWH (2, 3). Chronic immune activation and immune pathogenesis in HIV-1 infection are multifactorial and complex, with the bulk of long-term heightened responses deriving from increased proinflammatory bacterial lipopolysaccharide (LPS) translocation occurring across the gastrointestinal (GI) barrier (4, 5). The primary driver of GI barrier deficiency is thought to be infection-induced loss of tissue-resident CD4+ T cells at mucosal tissue sites (in particular barrier-protective Th17 cells), which does not fully revert upon ART treatment (6, 7). Dysfunctional lipid metabolism in GI-resident CD8+ T cells has also recently been implicated in disrupting gut epithelial barrier integrity (8). However, an increasing body of studies describes persistent expression of viral nucleic acids in chronically infected CD4+ T and myeloid cells in PWH, even under conditions of long-term ART (9–12). Some individuals on suppressive ART additionally display detectable viral transcripts and antigens resulting from the expression of defective proviruses (13–16). HIV-1 infects myeloid cells less efficiently than CD4+ T cells, but infected myeloid cells are detectable *in vivo* (17–22), in part because tissue-resident myeloid cell restriction by the dNTPase SAMHD1 may not be as universal as was originally thought (23, 24). Further, HIV-1-infected myeloid cells might be overrepresented at sites of tissue dysregulation and contribute to chronic immune activation (24). Besides proinflammatory cytokine production, sustained interferon secretion and expression of interferon-stimulated genes (ISGs) have also been linked to cellular dysfunction in the context of chronic HIV-1 infection (25–27). Therefore, persistent innate immune activation, especially in tissue-resident long-lived myeloid cells, which frequently serve as the gatekeepers of local innate immune responses and proinflammatory cytokine production, is likely to constitute a pathogenesis mechanism contributing to infection-associated comorbidities in PWH.

## HIV-1 NUCLEIC ACID SENSING EVENTS IN MYELOID CELLS

Research has linked innate immune system activation to viral nucleic acid sensing events early in the viral lifecycle, implicating the incoming single-stranded HIV-1 RNA genome or associated DNA reverse transcription (RT) products as pathogen-associated molecular patterns (PAMPs) (28–31). More recent studies have questioned this model and suggested that innate immune sensing of RT intermediates can be inhibited by the viral capsid lattice during early infection events, which physically shields and sequesters RT activities from the host cytosolic cyclic GMP-AMP synthase (cGAS)-stimulator of interferon genes (STING) pathway and orchestrates RT and integration steps (32–36). However, even without RT, activation of endosomal Toll-like receptors 7 and 8 (TLR7/8) by endocytosed virus particles, especially in plasmacytoid dendritic cells, has been documented as a mechanism of HIV-1-induced innate immune activation and is likely to contribute to systemic inflammation during acute infection/seroconversion and AIDS (31, 37, 38). It is unclear what role TLR-mediated innate immune activation realistically plays in the absence of viremia and robust viral replication. Indeed, the amount of viral nucleic acid PAMPs present in the target cell during early infection is most likely orders of magnitude lower than during productive replication. In contrast, cell-intrinsic innate immune responses to persistent viral RNA expression may have fewer barriers for chronic stimulation and play a more important role in long-term inflammation under conditions of viral suppression, where the overwhelming majority of persistently infected cells harbor defective proviruses incapable of virus particle production but theoretically capable of persistent transcription (2, 39–42) and induction of innate immune responses (43). Many proviruses remain competent for translation in addition to transcription, despite their intact or defective status (44, 45). Importantly, an increasing number of studies using ultrasensitive methods report detectable viral RNA and protein expression, especially in secondary lymphoid tissues (46–49), in ART-suppressed PWH. Protein-positive cells are repeatedly reported as a significantly smaller population within all viral RNA-positive cells (10, 50, 51).

## INNATE IMMUNE RESPONSES TO VIRAL TRANSCRIPTION

In contrast to innate responses to early steps in the viral life cycle, we and others have reported innate immune responses to a post-integration step, specifically intron-containing HIV-1 RNA expression in macrophages and DCs (52, 53). Innate immune activation, consisting of mitochondrial antiviral-signaling protein (MAVS)-dependent CD169 upregulation and IP-10 (CXCL10) secretion in macrophages, and maturation (evidenced by CD86 upregulation) and type I interferon secretion in DCs, was observed. Both immune activation phenotypes were abrogated by RT and integrase inhibitor treatment and were dependent on Rev/CRM1 intron-containing RNA export. Stable plasma membrane association of unspliced HIV-1 RNA was also associated with innate immune activation (52, 54). Notably, activation was maintained upon infection of a virus harboring a large deletion within *gag/pol*, implying that some defective viral transcripts may elicit innate responses. Co-culture of infected macrophages with CD4+ and CD8+ T cells resulted in upregulation of exhaustion-related markers including PD-1, CD160, and Lag-3 on T cells, suggesting deleterious functional consequences in both CD4+ and CD8+ T cells following contact with infected macrophages (52). Shortly after these studies, MDA5 was identified as the sensor responsible for detecting the Rev/CRM1-dependent export of HIV-1 unspliced RNA (usRNA) (55), and this was confirmed independently by us (56). MDA5 activates MAVS along with transcription factors IRF3/5/7, TRAF6 E3 ubiquitin ligase, and IKK-β kinase. Of the three IRFs, IRF5 had the greatest effect on HIV-1-induced innate immune responses and also was the most significantly upregulated in macrophages from older (age 50+) leukocyte donors compared to younger (under 35 years) donors (56). Interestingly, IRF5 activation is recognized as a key regulator of macrophage and specialized macrophage-like cell function, and it is reported to promote M1 polarization phenotypes and contribute to inflammation and pathogenesis in the brain, adipose tissue, and liver (57–59). MDA5-dependent responses, characterized predominantly by robust secretion of proinflammatory IP-10 and expression of ISGs, were greater in older donors despite greater viral particle secretion overall from younger donors and might contribute to infection-associated inflammation in aging PWH (56, 60).

Another report from our group described activation of the NLRP1 inflammasome in macrophages in response to cytoplasmic expression of HIV-1 intron-containing RNA, which led to IL-1β secretion and caspase-1-mediated death of infected and/or bystander cells in macrophages and microglia (61). Our study also reported the reactivity of cells expressing HIV-1 intron-containing RNA with antibodies specific for double-stranded RNA (dsRNA). This result suggests that single-stranded HIV-1 intron-containing RNAs may contain double-stranded motifs, which could explain their susceptibility to MDA5 and NLRP1, both of which reportedly sense dsRNA (62–64). NLRP1 has also been linked to translation stress and ribosomal collisions, which could be relevant to its activation by HIV-1 intron-containing RNA (65–67). Translation of unspliced HIV-1 RNAs is intricately regulated, with evidence indicating important roles of 5′ cap hypermethylation (68, 69) and virus-encoded internal ribosomal entry sites (IRES) (70–72), either of which could theoretically differentiate HIV-1 translation from host cell mRNA translation and allow for innate immune sensing.

While HIV-1 RNAs have been linked to diverse innate immune sensing events and induction of proinflammatory and antiviral responses, the molecular details and structural features of the specific RNA species responsible have yet to be clearly defined. For instance, RNA modifications by host cell methyltransferase enzymes have been described to repress sensing of HIV-1 RNA (28, 73). Over the last decade, it has become clear that HIV-1 generates two populations of full-length usRNA to control the complementary functions of genome packaging and Gag/Pol translation during replication, ultimately orchestrated by the infected host cell’s transcriptional machinery (74, 75). RNA polymerase II initiates variably on a conserved GGG tract in the 5′ long terminal repeat (LTR) to produce ^cap^3G (GGG-) and ^cap^1G (G-) usRNAs, which differ in their 5′ UTR structure and cytoplasmic function (76–79). The 5′ cap-exposed ^cap^3G usRNAs are efficiently translated by ribosomes to generate Gag and Gag-Pol polyproteins for virus particle assembly, while the 5′ cap-sequestered ^cap^1G usRNAs transit through the cytoplasm to the plasma membrane, dimerizing to be packaged as the viral genome (80–83). Our most recent study reported that MDA5 sensing of intron-containing transcripts is specific to ^cap^1G unspliced species (54). Macrophages infected with wild-type (^cap^1G- and ^cap^3G-expressing) or ^cap^1G-only virus secreted type I interferon and IP-10, while macrophages infected with ^cap^3G-only virus did not. Unspliced ^cap^1G usRNAs, compared to ^cap^3G usRNAs, were the most abundant usRNA species in macrophages and preferentially localized at the plasma membrane. Mutations that pushed ^cap^3G usRNAs to adopt a ^cap^1G-like fold characterized by the exposure of the dimerization-initiating “kissing loop” (also known as the dimerization initiation sequence or DIS) but retaining an exposed 5′ cap failed to rescue MDA5-mediated responses to ^cap^3G usRNA (54, 81). Also necessary for MDA5 sensing was Rev/CRM1 export: ^cap^1G usRNA exported via an NXF1-dependent pathway similar to cellular mRNAs by inclusion of retroviral constitutive transport elements was efficiently translated but not detected by MDA5, altogether pointing to a functional connection between escape from host cell translation machinery, efficiency of genome dimerization and packaging, and MDA5 sensing susceptibility. This intriguing finding suggests that infected myeloid cells that transcribe ^cap^1G unspliced HIV-1 RNA (whether intact or defective) and engage CRM1-mediated nuclear export pathways might contribute to persistent stimulation of innate immune responses.

## CONFLICTING MODELS OF HIV-1 PATHOGENESIS ON ART

**Fig 1:**
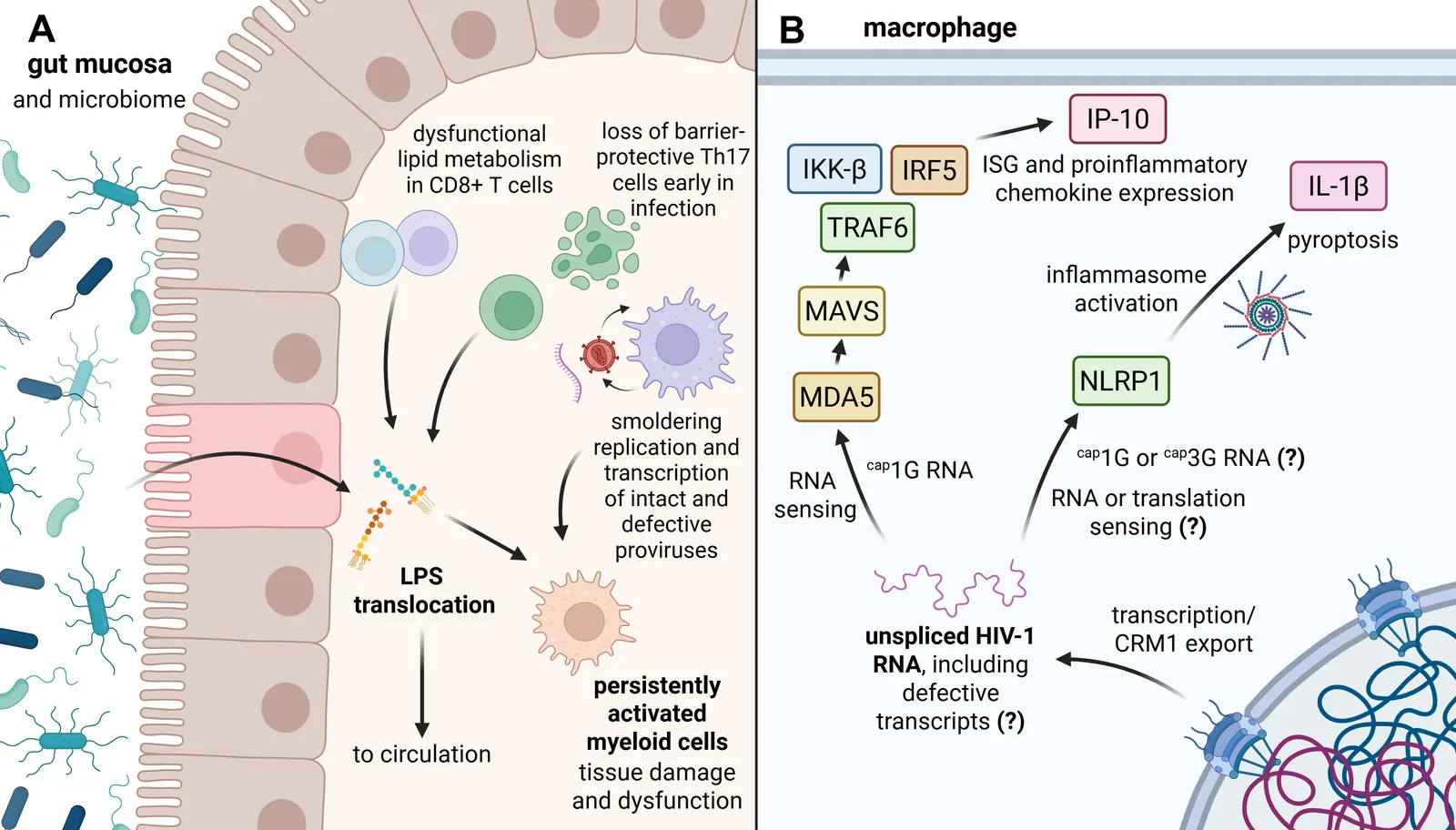
Mechanisms of nucleic acid-induced inflammation in HIV-1-infected tissue-resident myeloid cells. (**A**) Loss of protective CD4+ T cell subsets in the gut mucosa early in HIV-1 infection and metabolic dysfunction of resident CD8+ T cells contribute to barrier disruption and translocation of proinflammatory lipopolysaccharide (LPS) into the interstitium and circulation, which partially but not completely, reverts during suppressive ART. Chronic LPS stimulation could synergize with smoldering viral replication and innate immune sensing of HIV-1 transcription in tissue-resident myeloid cells, resulting in persistent activation of the myeloid compartment and tissue damage and dysfunction. (**B**) Interactions of HIV-1 nucleic acids with innate immune sensors in macrophages. After infection establishment, transcription and Rev/CRM1 nuclear export of full-length unspliced and probably some defective HIV-1 transcripts are sensed by MDA5 and NLRP1, resulting in proinflammatory cytokine and chemokine secretion and expression of interferon-stimulated genes (ISGs).

The role of persistent viral transcription in long-lived tissue-resident myeloid cells in chronic immune activation in PWH remains controversial given the diverse mechanisms of immune activation suggested to occur during chronic HIV-1 infection, notably gut barrier dysfunction. Reasons for this discrepancy include sampling difficulties in convincingly demonstrating persistent transcription in chronically infected myeloid cells at key tissue sites in PWH and a lack of suitable animal models that recapitulate HIV-1 infection in gut-resident macrophages and virus replication-induced alterations in intestinal barrier integrity, making direct mechanistic studies difficult. Chronic stimulation of infected tissue-resident macrophage populations with microbiome-derived factors could theoretically synergize with transcription-induced inflammation to promote long-term organ-specific pathogenesis in PWH (Fig. 1A). Ultimately, treatment approaches for residual HIV-1-related inflammation during ART treatment will be informed by the specific triggers implicated (i.e., the gut barrier and microbiome, residual viral antigens and nucleic acids, or both).

A common perspective in the field has held that immune responses to persistent transcription were unlikely, given that transcriptionally active infected cells harboring intact proviruses are strongly selected against over time by the adaptive immune response, and HIV-infected cells surviving years of ART are scarce in PWH (84, 85). However, the number of detectable RNA-positive cells in peripheral blood correlates with inflammatory markers and HIV-specific CD4+ and CD8+ T-cell counts in ART-suppressed PWH (10, 40, 86), though they increasingly display markers of immune exhaustion (10, 87), implying that persistent transcription and MHC-mediated presentation of HIV-1 antigens have a functionally meaningful effect on immune system constitution and homeostasis. Recent studies have also described non-suppressible viremia in PWH attributed to expansion of cells with replication-defective proviral clones (13–15). It remains unclear exactly which kinds of defective transcripts MDA5 or NLRP1 can sense (Fig. 1B), though our recent findings point to ^cap^1G 5′ UTR structure and Rev/CRM1 functionality as key and possibly minimal elements of MDA5 recognition, which could theoretically persist in some defective transcripts long-term. Importantly, however, Rev/CRM1 nuclear export is frequently compromised in infected cells surviving long-term due to inactivation of the Rev-response element (RRE) in defective proviruses (88). While Rev/CRM1 export is required for efficient genome packaging and activation of MDA5, NLRP1 activation is seemingly independent of the nuclear export pathway to some extent (54, 61, 89). A priority of future studies should be to further dissect MDA5 and NLRP1 activation resulting from chronic transcription of defective proviruses and probe the magnitude of persistent transcription in tissue-resident myeloid cells in PWH.
